# Carbon dioxide poisoning: a literature review of an often forgotten cause of intoxication in the emergency department

**DOI:** 10.1186/s12245-017-0142-y

**Published:** 2017-04-04

**Authors:** Kris Permentier, Steven Vercammen, Sylvia Soetaert, Christian Schellemans

**Affiliations:** 1 AZ St. Blasius, Dendermonde, Belgium; 2 Ghent University, Ghent, Belgium

**Keywords:** Carbon dioxide, Carbon dioxide intoxication, Dry ice, Intoxication

## Abstract

The goal of this article was to provide an overview of the literature available on carbon dioxide intoxication. Articles were included based on their focus on medical or physiological effects of carbon dioxide. Studies related to decompression sickness were excluded. Mechanisms of carbon dioxide poising (both as an asphyxiant and as a toxicant) were described. Our review suggested that precautions are needed when handling dry ice or while working in confined spaces. Pre-hospital responders also need to pay attention for the possible diagnosis of CO_2_ intoxication for their own safety. When confronted with a victim, he/she should be removed from the dangerous area as fast as possible and oxygen should be administered. Without adequate treatment, victims may show acute reduced cognitive performance, respiratory failure, and circulatory arrest. Therefore, carbon dioxide poisoning is a rare but not to miss diagnosis in the emergency department.

## Review

### Background

Carbon dioxide (CO_2_) is a product of combustion, fermentation, and respiration. In normal room air, carbon dioxide percentages are very low (around 0.04%). It is a colorless, odorless, and nonflammable gas that accumulates near the ground (CO_2_ is 1.5 times heavier than air). These characteristics explain why enclosed environments are vulnerable for CO_2_ buildup, displacing oxygen from the area [1]. The term “confined space hypoxic syndrome” has been proposed to describe confined space accidents occurring in water meter pits, tanks, holds of ships, mines, underground storage bins, and so forth, resulting from oxygen-deficient atmospheres [2, 3].

Studies conducted in the 1980s showed that there were 89 deaths per year in the USA alone, during work in confined spaces; 22% occur during rescue efforts [4]. A number has not decreased since. A recent study of the Occupational Safety and Health Administration (OSHA) in 2015 still estimated the number of deaths involving confined spaces to be around 90 per year. Unfortunately, the number of killed rescuers has risen to two thirds of those deaths [5].

Carbon dioxide does not only cause asphyxiation by hypoxia but also acts as a toxicant. At high concentrations, it has been showed to cause unconsciousness almost instantaneously and respiratory arrest within 1 min [6].

Other causes of carbon dioxide intoxication have been identified as well, such as dry ice. Dry ice undergoes sublimation (direct conversion from a solid state into a gas), and if it is warmed rapidly, large amounts of CO_2_ are generated, which is especially dangerous in closed environments [7–9]. Dry ice intoxication might be accidental [1, 7, 10, 11] or deliberately as several suicide cases have been described [8, 9]. Massive geothermal emissions have also been described as a possible cause of CO_2_ intoxications, though in these cases, a relation with other toxic gasses inhaled might not be excluded [12, 13]. We performed this literature review to understand the contribution of CO_2_ specifically to “confined space hypoxic syndrome,” and wanted to make physicians more aware of the condition.

### Methods

A literature review was performed where articles were sought in MEDLINE medical database via PubMed using the search terms: “dry ice poisoning,” “carbon dioxide poisoning,” “CO_2_ poisoning,” “carbon dioxide intoxication,” and “CO_2_ intoxication.” The references of the articles that were found were further evaluated to incorporate as much of the available literature as possible. Articles were included based on their focus on medical or physiological effects of carbon dioxide. Studies related to decompression sickness were excluded. Each article was independently evaluated by two of the authors. For all of the articles, an unanimous consensus among the authors was reached whether to include or exclude. In the end, a total of 19 articles were evaluated to be relevant to this topic and included in this review.

### Results

#### CO2 toxicity in animal models

Tests performed on mongrel dogs show the physiological effect of carbon dioxide on the body: after inhalation of a 50% CO_2_ and 50% air mixture, respiratory movement increased for about 2 min, and then, it decreased for 30–90 min. Hill and Flack showed that CO_2_ concentrations up to 35% have an exciting effect upon both circulation and respiration, but those beyond 35% are depressant upon them [6, 14]. The blood pressure (BP) decreased transiently during the increased respiratory movement and then rose again and maintained the original level for a while. The heart rate slowed slightly just after the gas mixture inhalation. It is believed that the initial BP depression with the decreased heart rate is due to the direct depressant effect of CO_2_ upon the heart and that the return of blood pressure to its original level was due to the rapid rise of PaCO_2_. After 30–90 min, the respiratory center was depressed, and hypotension occurred gradually or suddenly from reduced cardiac output, leading to an apnea and eventually to circulatory arrest [6].

In higher concentrations of CO_2_, unconsciousness occurred almost instantaneously and respiratory movement ceased in 1 min. After a few minutes of apnea, circulatory arrest was seen. These findings show that the cause of death in breathing high concentrations of CO_2_ is not the hypoxia but the intoxication of carbon dioxide [6].

#### CO_2_ toxicity in humans

Carbon dioxide at low concentration has little, if any, toxicological effects. At higher concentrations (>5%), it causes the development of hypercapnia and respiratory acidosis. Severe acidosis increases the effects of parasympathetic nervous activity, possibly by interfering the hydrolysis of acetylcholine by acetylcholinesterase, resulting in a depression of the respiration and the circulation [6]. Concentrations of more than 10% carbon dioxide may cause convulsions, coma, and death [1, 15]. CO_2_ levels of more than 30% act rapidly leading to loss of consciousness in seconds. This would explain why victims of accidental intoxications often do not act to resolve the situation (open a door, etc.) [7, 10, 16].

Studies have shown a wide variability of CO_2_ tolerance. Blood concentrations ranged between at least 0.055 and 0.085 atm. (41.8–64.6 mmHg) among subjects with symptoms, suggesting that a safe CO_2_ exposure level cannot be characterized by a single value [10]. Concentrations of fatal cases of carbon dioxide vary between 14.1 and 26% CO_2_ and an accompanying O_2_ level between 4.2 and 25% [1, 8, 11]. It was also determined that CO_2_ tolerance decreases with age (*p* < 0.0001) and suggested that smokers might have more tolerance due to habituation of higher CO_2_ levels in cigarette smoke [10, 16].

Effects of oxygen treatment have been studied on animal models, and both normal and high concentration oxygen have been recommended in the literature [16, 17]. Niu et al. showed in a study performed on Sprague Dawley rats who inhaled carbon dioxide gas that the levels of serum troponin I (CTNI), CK, serum potassium (K), and AST are lower afterwards when treated with hyperbaric oxygen therapy compared to other oxygen treatments (*p* < 0.05). Levels of serum sodium (Na) and Chloride (Cl) were higher in hyperbaric therapy (*p* < 0.05). There was no significant difference in pH, PO_2_, and PCO_2_ among all oxygen-therapy groups (*p >* 0.05); however, there were significantly less pathological changes in the lungs with hyperbaric therapy [17]. On the other hand, high concentrations of oxygen raise venous pO_2_ which reduces the solubility of carbon dioxide in the blood. With no changes in the metabolic conditions, this in turn causes pCO_2_ in the venous blood to rise. Due to this so-called Haldane effect, an initial increase of pCO_2_ in the bloodstream is to be expected when giving oxygen to a hypoxic carbon dioxide intoxicated person. It has also been suggested that due to high concentrations of oxygen, an increase in dead space is to be expected. P_ET_CO2 might therefore underestimate PaCO_2_. Other studies have found that there is no significant effect between administering normal levels of oxygen or hyperoxic gas [16, 17].

#### Diagnosing CO_2_ intoxication

Diagnosing CO_2_ intoxication is in the first place based on scene investigation and circumstances surrounding a victim [18]. Blood analysis will show increased carbon dioxide levels but is not always available pre-hospital [15]. Ischemic ECG changes (ST-segment changes and T-wave inversion) have also been described in several cases [19]. Victims might have burn wounds from contact with dry ice [15]. During massive geothermal emission events in Cameroon, cutaneous erythema and bullae have also been associated with coma states caused by exposure to carbon dioxide in the air on an unknown proportion of corpses and 161 (19%) survivors treated in a hospital. Though, it needs to be said that these were not reported in any other carbon dioxide case without contact to other acidic gasses [13].

#### Post-mortem diagnosis of carbon dioxide poisoning

Post-mortem identification of carbon dioxide intoxication might even be more difficult. External examination of the body is often unremarkable [1, 7]. Although, bruises and hematoma might be present from CPR or from other rescue efforts [10]. Blood analysis for CO_2_ content has only a limited diagnostic value, as CO_2_ rapidly accumulates after death [18]. Nevertheless, a case study in Japan showed higher levels of CO_2_ than those in the blood of deceased healthy persons. The levels found were similar to samples of fire victims, though there was no carbon monoxide (CO) found in this case (something that is usually found in fire victims) [8]. Analysis of lung gasses might also assist in determining the cause of death: an elevated CO_2_ content in lung gasses has been described after vacuum degassing of the lungs in an argon atmosphere and quadrupole-mass-spectrometry [10]. Victims might also show passive hyperemia of the internal organs, pleural petechiae, and edema with a moderate congestion of the lungs and the brains [1, 7, 10]. Due to the limited possibilities of proving lethal CO2 intoxications post-mortem, a close communication is needed among all of the involved parties, especially pre-hospital responders [9].

#### Management

When confronted with patients with carbon dioxide intoxication, the immediate removal of the casualty (and unprotected rescuers) from the toxic environment is needed [15]. Oxygen should be administered, and appropriate supportive care is advisable [15]. Relating to treatment, both normal and high concentration oxygen have been recommended in the literature, without a definite consensus being reached at the moment. Benefits of each approach have been discussed earlier within the topic “CO_2_ toxicity in humans” [16, 17].

## Conclusions

Carbon dioxide poisoning, having a role both as an asphyxiant and as a toxicant, is a rare but not to miss diagnosis. Special attention is needed for pre-hospital responders, who should stay alert for the possibility of a CO_2_ intoxication for their own safety, especially in cases involving dry ice or confined spaces. When confronted with a victim, he/she should be removed from the dangerous area as fast as possible and oxygen should be administered. Without adequate treatment, victims may show acute reduced cognitive performance, respiratory failure, and circulatory arrest.
